# A longevity society requires integrated palliative care models for historically excluded older people

**DOI:** 10.1016/S2666-7568(22)00031-9

**Published:** 2022-04-04

**Authors:** William E Rosa, Afsan Bhadelia, Felicia Marie Knaul, Jasmine L Travers, Nicholas Metheny, Terry Fulmer

**Affiliations:** Department of Psychiatry and Behavioral Sciences, Memorial Sloan Kettering Cancer Center, New York, NY 10022, USA; Department of Global Health and Population, Harvard TH Chan School of Public Health, Boston, MA, USA; Institute for Advanced Study of the Americas, University of Miami, Coral Gables, FL, USA; Institute for Advanced Study of the Americas, Miller School of Medicine, University of Miami, Coral Gables, FL, USA; Rory Meyers College of Nursing, New York University, New York, NY, USA; School of Nursing and Health Studies, University of Miami, Coral Gables, FL, USA; The John A Hartford Foundation, New York, NY, USA

Achieving a longevity society—one focused on the benefits of living longer and a healthy life expectancy—will require increased adoption of preventive health measures, equitable public health interventions, and the dismantling of ageist structures.^1^ Palliative care is a crucially overlooked component of a longevity society. The best evidence supports fully integrated palliative care models to ensure biopsychosocial support starting at the diagnosis of a life-limiting or life-threatening condition.^2^ Although the concepts of longevity and palliative care at first appear to be contradictory, access to palliative care is key to optimising the additional time that longevity affords, particularly for historically excluded populations who have poorer quality-of-life outcomes worldwide. In short, there is no longevity society without equitable palliative care access—and there will be no equity in alleviating serious health-related suffering until we amass the global policy and practice shifts needed to create and sustain inclusive, age-friendly health systems.

People aged 70 years and older account for 40% of patients in need of palliative care worldwide.^3^ Looking ahead, serious health-related suffering that could be relieved with palliative care is expected to increase by an estimated 183% by 2060, driven primarily by increased rates of noncommunicable diseases.^4^ Older people living in low-income and middle-income countries, where the share of the population older than 65 years is predicted to double by 2050,^5^ will face the compounded burden of serious health-related suffering at the intersection of ageism, poverty, increased prevalence of noncommunicable diseases, and little-to-no palliative care or age-friendly access to health services. However, fully integrated palliative care as a component of universal health coverage^2^ would be likely to stimulate the needed sociopolitical shifts towards a longevity economy^6^—one that actively encourages older people to participate in and contribute to society in accordance with their wishes while holistically addressing their needs through readily available, evidence-based symptom management and psychosocial care.

We must garner multi-sector support to develop and sustain age-friendly systems that strategically expand age-friendly health services by considering the “4Ms”:^7^ prioritising age-friendly Medication plans, preserving Mentation, and ensuring safe Mobility—all of which assist older people to do and achieve What Matters most in terms of their health goals, values, and preferences. Integrated palliative care will aid in cultivating both clinicians and systems grounded in an age-friendly ethic to ensure person-centred services throughout the serious-illness continuum and especially at the end of life. It is incumbent upon the health-care workforce to develop the communication competencies and community-oriented partnerships that will attune the delivery of palliative care to the cultural needs and experiences of all older people, especially those who are at greater risk of violence, abandonment, and mistreatment through the perpetuation of pervasive discriminatory structures. Structurally vulnerable populations that experience serious health-related suffering alongside trauma (historical or ongoing), social exclusion, and discrimination have complex palliative needs that must be met across the life course and throughout longevity.^8^

In addition to the consequences of poverty and ageism, many older people have minoritised identities (eg, racial, ethnic, and cultural) that render them actively and systematically excluded from accessing equitable palliative care. For instance, sexual and gender minorities might face homophobia, transphobia, social isolation and stigma, disenfranchised grief and bereavement, violence, and criminalisation.^9^ Indigenous populations frequently have unmet palliative care needs that exacerbate disparities, such as culturally unsafe care that disregards traditional healing practices, stymies accessibility, and exacerbates financial toxicity.^10^ Studies have also shown poorer pain and symptom management, lower rates of advance care planning, and lower utilisation of hospices among nursing home residents who identify as a racial or ethnic minority.^11^ Nursing homes in a number of countries were abandoned by policymakers at the beginning of COVID-19, leading to devastating consequences of social isolation for many residents, particularly for those in marginalised groups—a moral failing that integrated palliative care could help to correct and prevent during future public health crises. Moreover, current health systems are often not responsive to shifting cultural constructs and realities around ageing that could be addressed through palliative care. For example, among immigrant populations within culturally diverse societies, there are changing expectations of intergenerational support in ageing care and the myriad stressors of ageing in a new cultural context.

Integrated palliative care must be community-based, society-supported, and culturally safe to recentre historically excluded older people (panel). Publicly provided, universally accessible palliative care models can serve as levers for health equity, improved public health outcomes, and the global alleviation of serious health-related suffering. Integrated palliative care guided by the age-friendly health system movement, particularly for older people with minoritised identities, is a feasible and evidence-based approach to rehumanise health care systems. Every person deserves the promise of thriving into older age. Without equitable palliative care access, the longevity society will be a long time coming.

## Panel: Integrated palliative care at the intersection of a longevity society and age-friendly systems: recommendations for policy, academic, and clinical domains

- Reframe palliative care to support age-friendly lived environments and lived realities, and reorient services to account for social determinants of healthy ageing
- Promote public discourse and knowledge co-production on core features of a longevity society as they pertain to integrated and responsive palliative care
- Develop national, state, and local programmes that promote the use of hospice and palliative care, reduce associated stigma, and enhance health professional and public knowledge about palliative care
- Situate palliative care as a community-partnered and community-owned aspect of a longevity society through community-oriented policies at multiple levels
- Fully integrate hospice and palliative care into socialised and private insurance schemes and incentivise beneficiaries to utilise these services
- Improve access to mental health services and reduce disenfranchised grief by including mental health in existing hospice and palliative care delivery models
- Reduce ageism within palliative care by strengthening evidence-based anti-ageist training for health professionals
- Develop and implement training for clinicians working across the lifespan that promotes culturally safe, equitable care delivery
- Increase hours dedicated to palliative care in multidisciplinary health curricula
- Implement data collection processes to track and evaluate hospice and palliative care efforts that recentre marginalised groups
- Use community-based participatory research to engage stakeholder perspectives and priorities throughout empirical endeavours and palliative care efforts
- Integrate cultural safety as the dominant paradigm for working with older people with multiple minoritised identities who are experiencing serious health-related suffering
- Ensure health professionals feel equipped to provide inclusive and longevity-minded care to all older adults (eg, initiating hospice and palliative care conversations)
- Improve partnerships and communication among local hospice and palliative care delivery agencies about care needs to improve healthy life expectancy
- Develop hospice and palliative care communication material that is culturally inclusive, particularly accounting for culturally diverse perceptions of ageing and palliative care
